# Identification errors in camera-trap studies result in systematic population overestimation

**DOI:** 10.1038/s41598-020-63367-z

**Published:** 2020-04-14

**Authors:** Örjan Johansson, Gustaf Samelius, Ewa Wikberg, Guillaume Chapron, Charudutt Mishra, Matthew Low

**Affiliations:** 1 Grimsö Wildlife Research Station, Department of Ecology, Swedish University of Agricultural Sciences, SE-73091 Riddarhyttan, Sweden; 2 Snow Leopard Trust, 4649 Sunnyside Avenue North, Seattle, USA; 3 Nordens Ark, Åby säteri, 456 93 Hunnebostrand, Sweden; 4 Nature Conservation Foundation, 3076/5, IV Cross Gokulam Park, Mysore, India; 5 Department of Ecology, Swedish University of Agricultural Sciences, SE-75007 Uppsala, Sweden

**Keywords:** Conservation biology, Biodiversity

## Abstract

Reliable assessments of animal abundance are key for successful conservation of endangered species. For elusive animals with individually-unique markings, camera-trap surveys are a benchmark standard for estimating local and global population abundance. Central to the reliability of resulting abundance estimates is the assumption that individuals are accurately identified from photographic captures. To quantify the risk of individual misidentification and its impact on population abundance estimates we performed an experiment under controlled conditions in which 16 captive snow leopards (*Panthera uncia*) were camera-trapped on 40 occasions and eight observers independently identified individuals and recaptures. Observers misclassified 12.5% of all capture occasions, resulting in systematically inflated population abundance estimates on average by one third (mean ± SD = 35 ± 21%). Our results show that identifying individually-unique individuals from camera-trap photos may not be as reliable as previously believed, implying that elusive and endangered species could be less abundant than current estimates indicate.

## Introduction

**Figure 1:**
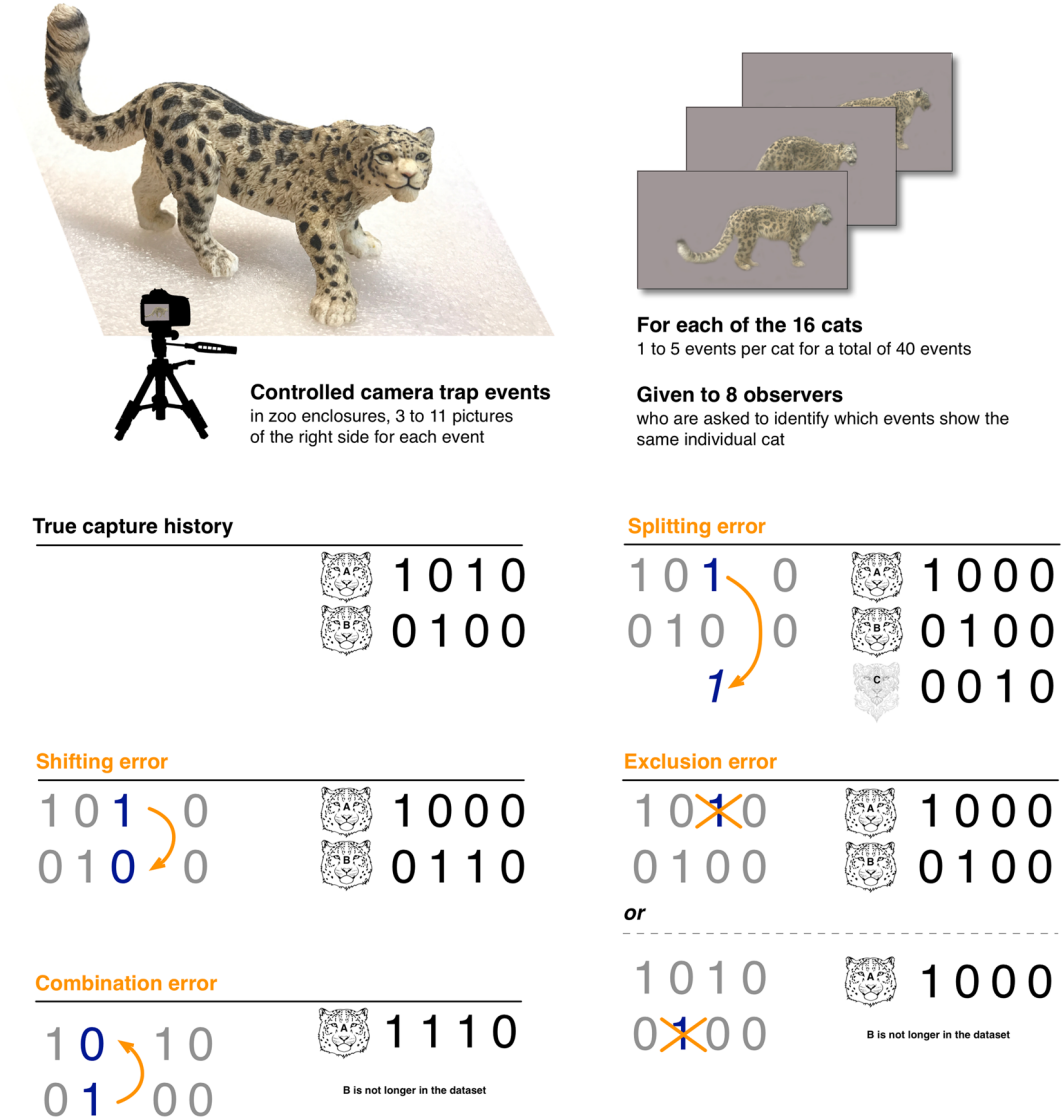
Conceptual figure of the experiment and influence of different errors on the structure of the capture histories (CH) in photograph-based population abundance estimation. Here, the true CH contains an individual (**A**) who was captured twice using a camera-trap and another individual (**B**) who was captured once. Capture-recapture methods use the number of individuals and the proportion of captures (1) and non-captures (0) to estimate the population abundance; thus anything influencing either of these factors will influence the population estimate. A shift error moves a capture event from one individual to another, but does not change the total number of individuals or the number of 1’s and 0’s (middle left). A combination error combines the captures from two individuals into one, reducing the number of individuals and the total number of zeros (lower left). A splitting error splits the captures from one individual into two and creates a “ghost” individual, increasing the number of individuals and the total number of 0’s (top right). A capture exclusion where identification is possible, is a form of identification error that changes a 1 to 0 in that individual’s CH (bottom right). Population abundance estimates will be underestimated by combinations and overestimated by splits. In a conventional capture-recapture framework, shifts will largely not affect population estimates. Exclusions may over- or underestimate the abundance, depending on whether they result in the loss of individuals from the CH or if they are non-random relative to individual identity (bottom right). The image was created in the software OmniGraffle 7 (https://www.omnigroup.com/omnigraffle).

When using photographic surveys (e.g. camera-trapping) to derive population abundance or demographic parameter estimates using capture-recapture analytical methods, it is critical that individuals are reliably and accurately identified from images to avoid estimation biases^1–4^ (Fig. 1). It is well recognized that misclassification of photographs may occur when identifying individuals using natural marks or patterns because of poor photographic quality^1,5^, if the variability in marking patterns is small^1,6,7^, or if patterns vary over time^3^. However, in terrestrial species with individually-unique natural markings such as stripes [e.g. tiger (*Panthera tigris*), wildebeest (*Connochaetes taurinus*)] or spots [e.g. cheetah (*Acinonyx jubatus*), snow leopard (*Panthera uncia*)], it is generally assumed that individuals are accurately identified^4,8–11^. This is despite there being almost no empirical evidence to verify this assumption because camera-trapping studies on species with individually-unique markings rarely report how identification was performed, the number of photographs that were unidentifiable, or if there was inter-observer heterogeneity in assigning identities^6,11–14^. Also, there is no baseline measure of classification error in these species because studies have not been undertaken to measure classification accuracy in a population of individuals with known identity. Thus, it is currently unknown how much observational uncertainty is associated with classifying images of species with individually-unique markings, and how this subsequently influences confidence in abundance estimates. This is surprising, considering that many of the species surveyed photographically are threatened or endangered, and accurate population and demographic estimates are critical to their conservation^10,13,15^.

Despite this, significant progress has been made in addressing the general issue of incorrect classification of wildlife ‘recaptures’ within the capture-recapture analytical framework. These methods include: (1) using multiple observers to help identify classification errors or estimate observation error^13^, (2) including spatial information to probabilistically resolve issues of incomplete identity through spatial capture-recapture models^16^ and spatial partial identity models^17^, and (3) resolving genotyping errors by using sample matching approaches for genetic mark-recapture studies^18–20^. However, regardless of these advances in wildlife studies, the degree to which their application would solve issues of photographic misclassification for animals with individually-unique markings is difficult to determine if the types of errors being made and the frequency of these errors are unknown.

Four types of error can occur when identifying individuals from photographs (Fig. 1). First, photographic captures of the same individual can be split into two (a splitting error that creates an additional ‘ghost’ animal). Second, captures of two individuals are combined into one, so that an animal not captured previously is erroneously believed to be a recapture (a combination error). Third, a photographic capture is shifted from one individual’s capture history to another (a shifting error that results from a splitting error from the first individual in conjunction with a combination error to the second individual). Finally, a photographic capture is not assigned to any capture history, and instead is excluded from classification despite it containing enough information for it to be reliably classified (an exclusion error; Fig. 1). Previous efforts to estimate misidentification rates from camera-trapping surveys have been restricted to species with low intra-specific variation, as a means of determining whether photo identification is feasible in these species^7,21,22^. Further, such studies have either compared the level of agreement between observers^21^ or estimated an overall error rate^22,23^. Animals with individually-unique markings (e.g. tigers or snow leopards) have been largely ignored in this regard, where the possibility of misidentification errors has been assumed to be negligible. However, it is important to understand exactly where and how often errors arise, because different types of misidentification will affect population estimates differently: splitting errors will systematically overestimate abundance^19^, combination errors will underestimate abundance^20^, shifting errors can introduce significant bias in estimates from spatial capture-recapture approaches, while exclusion errors could potentially inflate abundance estimates if they do not simultaneously remove some animals from the capture history^18^ or negatively bias abundance estimates if exclusions are not random with respect to individual identity and hence increase detection heterogeneity^19^ (Fig. 1). Therefore, the rates at which splitting errors, combination errors, shifting errors and false exclusions occur need to be quantified in all species where photographic identification is used for determining population abundance estimates^3^, especially those where conservation priorities may be informed by these estimates^24,25^.

**Figure 2:**
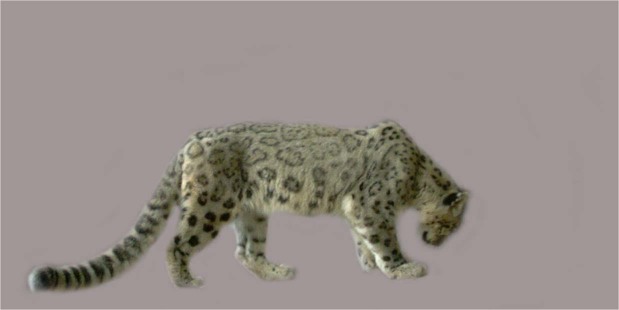
Example of photographs used in this study to assess identification errors in camera-trap photographs of snow leopards. Note the right side of the cat is visible and the background has been removed to prevent observers identifying the cat based on visual information from the background.

To this end we camera-trapped captive snow leopards (Figs. 1 and 2) in order to quantify: (1) how often observers correctly assigned the identities of individuals to photographs and the relative proportion of splitting, combination, shifting and false exclusion errors, and (2) how these errors have potential to translate into biases in population abundance estimates. We discuss the implications of our findings for photograph-based survey techniques to derive population abundance estimates in snow leopards and other conservation critical species.

## Results

### Misclassification errors

12.5% of capture events (hereafter ‘events’) were incorrectly classified, with the majority of mistakes coming from splitting errors (total splitting error probability = 0.111) rather than combination errors (total combination error probability = 0.037; see Table 2 for details). This pattern was similar for both experts and non-experts (Tables 1–3). Experts were generally less likely to make errors than non-experts, but still had a 9.9% probability of misclassifying an event (versus 14.6% for non-experts; Table 2), resulting in an average 2.5 ghost individuals created by splitting errors per expert’s capture history (versus 3.2 for non-experts; Tables 1 and 3). This ghost creation was as high as 31% of the true population (5 ghosts) for non-experts and 25% (4 ghosts) for experts (Table 1).

**Table 1:** The types of identification errors and the number of individuals added to or lost from the capture history for each observer (Obs1–4 are non-experts and 5–8 are experts).

	Exclude	Split	Shift	Combine	Individuals lost in excluded CEs	Individuals lost in combination errors	Ghosts created in split errors	Number of individuals identified (true)
Obs1	4	5	5	1	1	2	5	18 (15)
Obs2	12	2	0	0	3	0	2	15 (13)
Obs3	0	2	0	0	0	0	2	18 (16)
Obs4	1	4	1	0	0	0	4	20 (16)
Obs5	4	3	0	0	1	0	3	18 (15)
Obs6	1	4	0	0	0	0	4	20 (16)
Obs7	2	0	1	0	1	0	0	15 (15)
Obs8	1	3	0	3	0	1	3	18 (16)

Exclude = capture events (CE) removed from classification for being too difficult to classify, split = CEs split from one individual to create two individuals, combine = CEs combined from two individuals into one individual, shift = CEs split from one individual and combined with another. These errors result in the loss of individuals when: (1) they are not considered because all CEs containing their photos were excluded [here they are not present in the capture history], or (2) they are combined with another individual [here they remain in the capture history, but are misclassified]. Errors also result in false individuals being created (ghosts) from splitting errors. Thus the ‘number of individuals identified’ in a capture history can have three meanings: (1) how many unique animals the observer thinks they saw and classified; this is the number of rows in the capture history and equals: the true number photographed – individuals lost (exclusion or combination) + ghosts created, (2) how many unique animals the observer actually saw and classified (true); this equals number of animals recorded in the capture history (true number photographed – individuals lost through CE exclusion), and (3) in a very broad sense it could be interpreted to mean the population abundance estimate, since the capture history also contains information about animals that were not seen (see Table 3).

**Table 2:** Probabilities of identification errors while classifying each set of camera-trap photographs (capture event (CE) folders) of snow leopards (estimates are the mean ± SD of the posterior distribution of the expected mean error probability from Bayesian binomial models described in Appendix S1).

Error	Overall	Non-expert	Expert	P(Non> Expert)
Split	0.091 ± 0.016	0.098 ± 0.025	0.078 ± 0.021	0.709
Combine	0.016 ± 0.007	0.007 ± 0.007	0.019 ± 0.011	0.138
Shift	0.023 ± 0.008	0.042 ± 0.016	0.002 ± 0.001	1
Total split	0.111 ± 0.011	0.139 ± 0.028	0.078 ± 0.022	0.957
Total combine	0.037 ± 0.011	0.049 ± 0.017	0.019 ± 0.011	0.918
Exclude	0.087 ± 0.016	0.119 ± 0.027	0.053 ± 0.018	0.981
Exclude*	0.049 ± 0.013	0.044 ± 0.019	0.053 ± 0.018	0.344
CE error	0.125 ± 0.019	0.146 ± 0.029	0.099 ± 0.024	0.895
CE error +	0.208 ± 0.023	0.264 ± 0.036	0.151 ± 0.028	0.994
CE error* +	0.174 ± 0.023	0.191 ± 0.034	0.151 ± 0.028	0.782

Estimates are presented for the 8 observers (overall) and also divided according to their previous experience in snow leopard photo classification (non-expert vs. expert) with the probability that non-experts have greater errors than experts [P(non > expert) derived from the posterior distribution of the difference between observers]. Total split and total combine add the shift estimate to the split and combine estimates, respectively, since shifts involve a split and combination error. Capture event error (CE) relates to the total probability of a capture event being misclassified: this is presented for classification errors only (CE error) and when exclusions are considered as a classification error (CE error+). Because observer 2 excluded 30% of all capture events, some estimates are also presented where observer 2 has been removed from the analysis (*).

**Table 3:** Comparison of the structure of the capture histories (CH) derived from classification of snow leopard images by eight observers (Obs 1–4 were non-experts, Obs 5–8 were experts) with the true capture history (TRUE).

	CH structure					Population estimate	Bias in population estimate	
	5	4	3	2	1		True	Remaining
TRUE	1	2	5	4	4	16.6 ± 0.9	+3.7%	+3.7%
Obs1	0	2	3	6	7	20.3 ± 2.0	+27%	+35%
Obs 2	1	1	1	4	8	23.9 ± 7.2	+49%	+84%
Obs 3	1	2	3	6	6	21.0 ± 2.8	+31%	+31%
Obs 4	0	2	2	9	7	22.8 ± 2.3	+42%	+42%
Obs 5	1	1	3	5	8	23.5 ± 4.5	+47%	+56%
Obs 6	0	3	2	6	9	22.8 ± 2.3	+42%	+42%
Obs 7	2	0	5	5	3	16.1 ± 1.3	+0.6%	+7%
Obs 8	3	0	2	5	8	23.2 ± 3.9	+45%	+45%

The CHs were based on 5 sampling occasions; CH structure shows how many times each snow leopard individual was seen (where the ‘5’ column indicates an individual was recorded in 5 capture events, and the ‘1’ column indicates an individual was only identified by the observer once). Based on each observer’s CH a population abundance estimate was derived using a closed capture-recapture model (mean ± SD; see methods). The bias in the mean estimate for the population is shown relative to the true population size (n = 16) and also relative to the number of unique individuals remaining in each observers’ CH after accounting for animals removed from consideration because of capture event exclusion (for observers 1, 2, 5 & 7 the number of unique individuals assessed was n = 15, 13, 15 & 15 respectively; see Table 1).

### Exclusion errors

There was an 8.7% probability that an event would be excluded from classification, with non-experts having double the probability of excluding than experts (11.9% versus 5.3%; Table 1). However, one non-expert (observer 2) excluded 12/40 events (30%), which was substantially more than all other observers; if that observer was removed, then exclusion rates were roughly the same for non-experts and experts (4.4% vs 5.3%; Tables 1 and 2).

There was no evidence that events that were more likely to be excluded by some observers were subsequently more likely to be misclassified by the remaining observers who attempted classification (though the uncertainty around the estimates was large and such an effect may still exist without being detectable; Supplementary Fig. S1). The two events that were most commonly excluded (#25 & #31 were each excluded by four observers) were correctly classified by the remaining four observers. The events that were most commonly misclassified (#22 was misclassified by 7 of the 8 observers, #40 misclassified by 4 observers, and #24 & #38 misclassified by 3 observers) were either never excluded (#40 & #38) or were only excluded by the non-expert who excluded 30% of his/her events. This strongly suggests that our assumption of all events being classifiable was correct, and that all exclusions in this study can be considered as exclusion errors. Thus, if exclusions are included when calculating the probability of an error (i.e. misclassification + exclusion), the probability of experts making an event error was 15.1% and non-experts 26.4% (or 19.1% if observer 2 is removed). It should also be noted that, for this analysis, individual cats needed to be included as a random factor on the model’s intercept to account for overdispersion (Appendix S3); this ‘misclassification heterogeneity’ indicates that some individuals were more likely to be misclassified than others regardless of exclusion rates, clearly suggesting that there were individual differences in spot patterns of the snow leopards that made some easier to identify than others. This would indicate that exclusion and misclassification errors are likely non-random with respect to individuals.

### How errors affect capture histories and population estimates

Across all observers, there was an 11% probability that a split would occur, with the majority of these (81%) resulting in a ghost. For experts, almost all splits (95%) yielded ghosts because experts rarely made a ‘shift’ error (Tables 1 and 2), while for non-experts only 73% of splits yielded ghosts (i.e. because 27% of their splits arose within a ‘shifting’ error). Because splitting errors were always more common than combination errors, all observers created capture histories that contained more animals than the true capture history, and individuals had smaller numbers of recaptures (Table 3). This inflated population estimates over the true population size (n = 16) for experts and non-experts (mean inflation +33% versus +37% respectively) and ranged up to ~50% population overestimation (Table 3).

**Figure 3:**
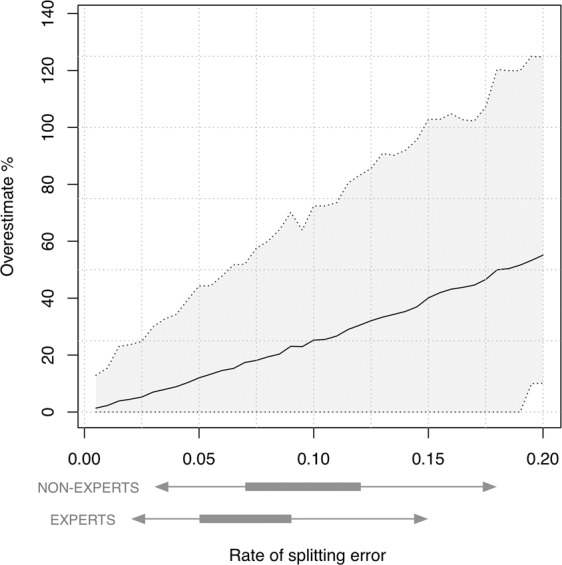
How different rates of ghost-producing splitting errors (0.05–0.20) affect the population abundance estimate, based on camera-trapping data from a real-world snow leopard study in Mongolia^21^ (where the population size = 12, capture occasions = 16, capture probability = 0.16). Estimates are derived from 1000 simulations at each error rate; the solid line is the median error and the grey shading the 95% quantile range. This is shown relative to the expected credible range of splitting errors (50% and 95% CIs as thick and thin lines respectively), from expert and non-expert observers (generated from the binomial likelihood of the model in Appendix S1; for splitting errors that create new individuals).

The two simulations clearly show that the potential impact of splitting errors on abundance estimates depends on three factors: the number of encounter occasions, the probability of detection and the number of splitting errors (Fig. 3; Supplementary Table S2). Based on a population of 12 individuals with 16 capture occasions and a capture probability at each occasion of 0.16 (similar to the most extensive field study to date, see^26^), a 10% splitting error leads to an average 25% overestimate of the true population size (with overestimates as high as 125% within the 95% confidence range; Fig. 3). When examining ranges of capture occasions (7–30) and capture probabilities at each occasion (0.1–0.4), a single splitting error could add anywhere from 1 to 8 additional animals to the true abundance estimate of 16 (population overestimated by 6–50%). Here, fewer capture occasions and lower capture probabilities result in the largest impact of splitting errors (Supplementary Table S2). The effect of each additional splitting error magnifies the error on the abundance estimate because the additional zeros it adds to the encounter history creates an increasingly smaller estimate of the capture probability (Fig. 3; Supplementary Table S2; Appendix S4).

## Discussion

Regardless of the extent and sophistication of the survey methods or analyses used to determine population abundance from camera-trapping data, incorrect classification creates false capture histories and biased estimates^1–4^. Until now it has been generally assumed that because some species have individually-unique markings (e.g. tiger stripes or cheetah spots), these allow researchers to correctly identify individuals upon re-encounter^9–11,26^. In an ideal situation this may be true; however, observation error is common in ecology even in situations where one might expect these errors to be small or absent^27^. Here we show that not only do experienced observers make mistakes, but in our experiment, these mistakes were reasonably common (one observation out of ten was misclassified), implying that they may impact population abundance estimation in real-world situations. These results raise questions regarding the breadth and magnitude of these biases in current field population estimates based on camera-trapping data and how this observational uncertainty (if present) can be reduced or accounted for in estimates. Our point here is not to recommend discarding camera trap studies, but instead to stress the need for additional rigor when identifying individuals from camera-trap photos to ensure that the derived estimates are as least biased as possible (this is similar to the issue of genotyping errors that has been a major focus in the non-invasive genetic capture-recapture literature^18–20^). Our study shows that the implicit assumption of individually-unique animals being always correctly identified is unlikely to be true.

Camera-trap surveys yield a number of capture events where individuals are either seen (1) or not seen (0) at each sampling occasion. In essence, population abundance estimates are derived from a capture–recapture framework where the number of individuals encountered during the survey is divided by the overall capture probability^28^. Since capture probability is a function of the number of animal detection over total capture occasions (i.e. the ratio of 1’s and 0’s in the capture history), anything that changes this ratio will affect the population abundance estimate. Thus, when observers make more splitting errors than combination errors, this inflates population abundance estimates not only via an increase in the number of individuals identified (ghosts added), but also by reducing the capture probability through the addition of zeros to the capture history (Fig. 1; Supplementary Table S2; Supplementary Appendix S5). In addition, excluded capture events (false exclusions) replace a ‘capture’ (1) with a ‘not observed’ (0), further reducing the 1:0 ratio. This can impact on abundance estimates in one of three ways: (i) the addition of ‘missing data’ will decrease the precision of estimates, (ii) missed recapture events inflate mark-recapture abundance estimates^18^, or (iii) if exclusions are non-random (as our results suggest) then the resulting inflation of recapture heterogeneity could negatively bias abundance estimates^20^. ‘Shift’ errors (split + combine) neither change the number of capture events nor the number of individuals identified. However, in multi-year studies, shifts could complicate future identification because the identification key for these ‘misidentified’ individuals will contain multiple different individuals. Similarly, in a spatial capture-recapture framework, shifts may introduce large spatial recaptures that positively bias the spatial scale parameter and negatively bias the detection parameter: this has the potential to substantially influence the abundance estimate.

In our study, splitting errors swamped any impacts of combination errors and led to population abundance estimates from experts being inflated by an average of 33% above the true population size. Subsequent simulations demonstrated that the impact of splitting errors was dependent on the number of capture occasions and capture probability at each event, with these effects becoming increasingly influential with each additional ‘ghost’ individual and quickly leading to population abundance overestimated by up to 50% (Fig. 3; Supplementary Table S2). This shows that the impact of these splitting errors on population abundance can be partly mitigated by increasing the number of capture occasions (although we have not explored the impact of this for spatial capture-recapture data). However, since total splitting errors are based on the number of classifications attempted (i.e. 1 split per 10 classifications in our study), any increase in the number of capture occasions needs to not only consider limiting the effect of each splitting error on population abundance estimates, but also limiting the total number of splitting errors. More quantitative investigation of the interplay between impact of splitting errors and number of capture occasions is needed.

Experts made one-third fewer classification errors than non-experts, and fewer false exclusions (although not if non-expert 2 was removed) cf.^22,29^. There is an urgent need to understand what type of ‘experience’ experts need in order to decrease classification errors or how targeted training could help reduce such errors. As a first step to achieve this, the photographs and capture events used in this study have been incorporated into an online training tool (camtraining.globalsnowleopard.org) where observers can practice identifying snow leopards and evaluate their risk of making different types of error. If similar efforts are developed for other species, individual experts could be trained to minimize their own classification errors, or at least to be more aware of the magnitude of the issue. These error rates can be collected from these training tools and subsequently used for incorporating observational error into the modelling framework, or to determine which advances in capture-recapture methodology (e.g. spatial capture-recapture methods) would likely reduce their impacts. In addition, if the number of excluded capture events and between-observer variation is reported for specific studies, potential bias and uncertainties in population abundance estimates could be more easily accounted for. A special emphasis on assessing the valid existence of new animals appearing in capture histories might also help reduce error propagation of ‘ghosts’ into abundance estimates, in much the same way that genetic capture-recapture methods have worked to minimize the risk of misclassification arising from genotyping errors^18–20^. Future approaches to solving some of these issues will likely rely on software classification using automated image recognition algorithms such as deep convolutional neural networks^30^; however, these are likely to suffer from some of the issues we describe here, and their errors will similarly need to be quantified.

The primary question our study raises is: to what extent does misclassification of camera-trap photos inflate current population estimates of wild felids and other species that rely on similar technologies? It appears that the possibility to inflate population estimates due to identification errors can be substantial, with our controlled condition experiment reporting an inflation of approximately 35% on average. Future studies need to examine observational error in this and other species to understand exactly how far the problem extends, and what methods are most effective at minimizing these errors.

This issue is not a trivial point of simply methodological interest. Population sizes and trends are the central parameters for all conservation and management decisions. Thus, if survey methods yielding these parameters are systematically biased, or are more uncertain than acknowledged, conservation decision making will suffer as a result. For example, the snow leopard was recently down-listed from Endangered to Vulnerable by the IUCN, with this being largely based on camera-trapping studies for population estimation and validation of other methods^31^. While previous research has shown that the size and types of areas surveyed for this assessment may overestimate density up to five-fold^32^, our study brings to light an additional source of potential uncertainty in the global population estimate. Because most felid species whose populations have been estimated by camera-trapping studies are threatened with extinction, our findings have potentially serious implications for other species with individual specific markings, such as tigers, leopards (*Panthera pardus*) and cheetahs, whose population abundances are based on similar survey techniques. Until studies on these species have been undertaken to quantify classification error rates and their impacts on population estimation, we recommend caution when judging current population estimates or inferring population responses to conservation actions. If our results turn out to be generalizable to field conditions and additional taxa, the populations of some threatened species may be smaller and therefore closer to extinction than currently believed.

## Methods

### Snow leopard photographic captures

We deployed one camera-trap (Reconyx HC-500, Reconyx, Holmen, USA) per snow leopard enclosure in seven European zoos (Helsinki and Ätheri Zoos in Finland, Kolmården Zoo, Nordens Ark and Orsa Bear Park in Sweden, and Köln and Wuppertal Zoos in Germany) from February to October 2012. The cameras were installed three to seven meters from trails that the snow leopards frequently used, to achieve similar photographic quality as is commonly gathered in the field (Fig. 2). We programmed the cameras to take five photographs on each trigger, with an interval of 0.5 seconds and no time lapse between triggers (same setup as typical field studies, e.g.^26^). Photographs were taken in the resolution 1080p. Only one snow leopard at a time was allowed in the enclosure when the camera-trap was active to ensure known identity of the individual in the photographs. In total, 16 adult snow leopards were photographed, which can be compared to a typical snow leopard data set from the field where 6 to 20 individuals photographed over a single sampling session have been reported e.g.^5,9,26^.

We created a photographic library containing 40 capture events, where each event contained a series of consecutive photographs from one of the 16 individual snow leopards. Each individual snow leopard was represented in one to five events (representing a range of recaptures across five sampling occasions) and the number of photographs within each event ranged from three to eleven to simulate a typical capture event (Supplementary Table S1; Fig. 1). Snow leopards have asymmetrical pelage patterns, similar to other spotted cats. This means that patterns on the animal’s left-hand side are different from those on the right-hand side. Criteria for inclusion in the library were: (i) the right-hand side of the snow leopard was displayed in at least one of the photographs, and (ii) combined with each other, the photographs showed enough of the animal’s side and were of sufficient quality to allow for individual identification. To ensure that background features could not be used to help the identification of animals, the background of all photographs was blurred using photo-editing software (Fig. 2).

### Individual identification and types of error

We asked eight observers to independently identify snow leopard individuals from the 40 events by examining distinct spot and rosette patterns (Table 1; Fig. 2). Four observers were researchers with previous experience in identifying snow leopards from camera-trap photographs (‘experts’), with three of these having authored peer-reviewed papers involving abundance estimation from camera-traps. The other four observers had experience of snow leopards in captivity but not in camera-trap photographic identification (‘non-experts’). The observers performed their work independently (for the observer protocol, see Appendix S1). If an observer felt they could not reliably identify an individual from the photographs in an event, it was excluded from further classification. This reduced the number of events for some observers (<40) and in some cases the total number of individuals if all events containing a given individual were excluded (Table 1; Supplementary Table S1; Fig. 1).

To calculate the probabilities of different identification errors, we evaluated if observers correctly classified each event by scoring it as correct or incorrect (Table 1; Supplementary Table S1). Incorrectly classified events were further scored into the following categories: (1) ‘split’, meaning the event was incorrectly split from other events containing the same individual and placed by itself, thereby creating a new individual, (2) ‘combine’, where the event(s) from an individual was combined with another individual, resulting in the loss of the focal individual (with one exception, this occurred with individuals that were represented by only one event), (3) ‘shift’, meaning the event was incorrectly split from other events containing the same individual and added to another individual’s set of events but this did not result in loss of the individual (i.e. a split + combine), or (4) ‘exclude’ because the snow leopard was deemed unidentifiable. Exclusion of an event where identification is possible is a form of identification error that affects capture-recapture calculations (by placing a 0 instead of a 1 at that (re)capture event; Fig. 1); thus we included it in our analyses for assessing the total number of event errors in addition to estimating its probability. Splits are single errors that create new individuals (sometimes referred to as ‘ghosts’) from previously known individuals. Combines are single combination errors that occur when a previously-unknown individual is misidentified as a known individual (in the genetic capture-recapture literature this is also known as the ‘shadow effect’^20^); these reduce the number of individuals in the sample (lost). Shifts consist of two errors, a split from one individual and a combination with another. Shifts do not affect the number of individuals identified but result in erroneous identification keys that can have major implications for spatial capture-recapture methods and increase the measure of capture heterogeneity. It is important to understand what these different errors represent when estimating the probability of a splitting or combination error. Total splitting errors are thus the number of splits + shifts; while total combination errors are the number of combines + shifts (for summary see Fig. 1 and Table 1). We present separate error estimates for experts and non-experts to highlight conditions where the two groups diverge and help the interpretation of the general estimates from all observers.

### Estimating observer misclassification & exclusion errors

We modelled each of the error categories (i.e. split, combine, shift, exclusion) as well as specific combinations of error categories (i.e. splitting, combination and total errors) using a logistic regression model (binomial likelihood) in a Bayesian framework run in JAGS^33^ within R^34^. This method allowed us to calculate the probability of each type of error occurring, based on the total number of events categorized by each observer (number of binomial draws, Appendix S2). Thus, for the exclusion category we consider all events as having the potential to be excluded; however for the split, shift and combine categories, we only considered the number of events that remained after the excluded capture events had been removed. We derived probability estimates not only for the general error rates, but also separate estimates for the two observer types: experts and non-experts. The advantage of using Bayesian models for these analyses was that we could directly calculate the probability that non-experts had greater exclusion, splitting or combination error rates than the experts. These resulting posterior distributions represented the difference between the two groups; thus, the proportion of the resulting posterior distribution that was above zero was the probability that non-experts were more likely to make errors than experts (the closer the proportion of the posterior distribution was to 0.5, the more likely there was no difference between the groups). We could also simulate expected values directly from the model’s likelihood function to generate the expected range of error rates from different observers in each category (see Appendix S3). For all models we used minimally-informative priors and ran a MCMC for 10 000 iterations after the chain convergence had been reached (see Appendix S3).

### Estimating capture event exclusion and misclassification

We allowed the observers to exclude capture events from classification if they felt they were not confident enough to make a decision regarding the identity of an animal, as would occur in a study of images captured in the field. We allowed this option despite us creating the events with what we believed were images that could reliably classify the individual. Thus, if exclusions occurred, we hoped to gain insights into how event exclusion may be interpreted as another source of observation error. To examine whether our assumption that all 40 events were possible to identify was correct (i.e. exclusions were ‘false exclusions’), and to better understand the nature of why events might be excluded in a field study, we compared misclassification rates and exclusion rates for the eight observers for each of the 40 events. If exclusions were in fact ‘true exclusions’ because they could not be reliably matched, then we expected higher misclassifications of those events when observers did attempt to classify them.

We modelled the number of misclassification errors for individual events as a function of three factors: (1) the number of times the events were excluded by the eight observers (range 0–4; the explanatory variable we were most interested in), (2) the total number of events that belonged to that individual cat if all events were assigned correctly (range 1–5) and (3) the individual cat identity. The model had a binomial Bayesian hierarchical structure with the number of trials (max = 8) adjusted based on the number of events excluded (i.e. only events that were classified in some way could be misclassified; Appendix S3). The total number of events belonging to each individual was included to control for the possibility that correctly classifying an event may be related to the total number of events linked to each individual: for example, a cat with a single event can only be combined with another cat, whereas a cat with multiple events can be split or shifted. Also, a cat with multiple events has more reference material, so the probability of making classification mistakes may be lower than for a cat with fewer events. Individual cat ID was included as a random effect on the intercept to account for the possibility of additional variation because some cats were more difficult to classify than others (independent of the decision to exclude or not; see Appendix S3).

### Impacts of splitting errors on population estimates

From each observer’s capture history we estimated the population size using a closed population capture-recapture estimation method [closedp() function from the R package ‘Rcapture’^35^] (Appendix S4). We used AIC to choose the highest ranking model’s population abundance estimate and compared this to the true number of animals in the population (n = 16) and also to the number of animals that were classified by each observer given that they may have excluded individuals from consideration (n ≤ 16).

To see the general effect of how misclassification of individual identities, and specifically splitting errors, within the capture history can influence population abundance estimates, we simulated snow leopard capture histories based on varying the number of capture occasions, capture probability and number of splitting errors. Because misclassification always created a net number of splitting errors (splits > combines; Table 1), this approach allowed us to investigate the general effect of how net misclassification of individual identities within the capture history influences population abundance estimates (Appendix S4 and S5). First, we simulated 1000 snow leopard capture histories based on the number of capture occasions and capture probability from our snow leopard field study in Mongolia^26^ and subsequently introduced splitting errors with a probability ranging from 0.5% to 25%. This was to examine how the observed rates of misidentification (between the different observers) translate into errors of population abundance estimates in our study population. Second, we simulated 1000 snow leopard capture histories for each of a variety of capture occasions and capture probabilities while introducing splitting errors (up to 5) to examine more generally how the creation of new (ghost) individual identities to the capture history influences population abundance estimates (Appendix S4 and S5).

## Supplementary Information

Supplementary Information.

## Data Availability

All data is available at camtraining.globalsnowleopard.org.
