# Supplementary material for: Identification errors in camera-trap studies result in systematic population overestimation
Source: Sci Rep. 2020 Apr 14;10:6393. doi: 10.1038/s41598-020-63367-z (PMC7156508; doi:10.1038/s41598-020-63367-z)
Supplement: Supplementary file 1 — Supplementary Information. [file 41598_2020_63367_MOESM1_ESM.docx]

Supplementary Material:

**Identification errors in camera-trap studies result in systematic population overestimation**

Örjan Johansson, Gustaf Samelius, Ewa Wikberg, Guillaume Chapron, Charudutt Mishra, Matthew Low

Supplementary Tables S1 & S2

Supplementary Fig. S1

Appendix S1 – protocol given to observers in this study

Appendices S2, S3, S4 & S5 – modelling details

References

**Supplementary Table S1**. Number of capture events (total = 40; range 1-5 for each individual, representing sightings during capture-recapture occasions) with photographic data for each individual snow leopard (n = 16) and the number of unique photographs of the individual snow leopard contained in each event..

| Snow leopard | Number of capture events | Photographs in each capture event |
| --- | --- | --- |
| L | 5 | 4, 5, 7, 10, 4 |
| A | 4 | 8, 8, 6, 8 |
| K | 4 | 5, 5, 6, 11 |
| B | 3 | 3, 5, 3 |
| D | 3 | 4, 6, 3 |
| M | 3 | 8, 5, 9 |
| F | 3 | 7, 3, 5 |
| N | 3 | 4, 9, 5 |
| P | 2 | 5, 6 |
| I | 2 | 4, 4 |
| J | 2 | 3, 7 |
| C | 2 | 3, 6 |
| E | 1 | 6 |
| G | 1 | 7 |
| H | 1 | 3 |
| O | 1 | 7 |

**Supplementary Table S2**. Population abundance estimates (mean ± SD and the 95% quantile range) from closed capture-recapture models based on 1000 simulated capture histories for a range of capture history parameters (capture-recapture probability [‘capture’] ranging from 0.1-0.4 per capture occasion; and number of capture-recapture occasions [‘occasions’] ranging from 7-30), and splitting error misclassifications in the capture history (ranging from 0-5). When the number of occasions was small (i.e. 7), we show all variants of the capture probabilities we tested to demonstrate that the results are highly sensitive to the capture probability (and hence splitting errors) when the number of occasions is small. When the number of occasions was much higher (15 or 30) in the simulations, we only show two examples of capture probabilities to highlight the fact that the effect of splitting errors is less pronounced. In all cases the true population size that the capture histories were simulated from = 16.

|  | | Rate of splitting error | | | | | |
| --- | --- | --- | --- | --- | --- | --- | --- |
|  | | 0 split | 1 split | 2 split | 3 split | 4 split | 5 split |
| *Occasions = 7* | |  |  |  |  |  |  |
|  | Capture =0.1 | 14.5 ± 6.1 (9 - 29) | 23.2 ± 13 (13 – 40) | 49.2 ± 45 (16 – 58) | 57.7 ± 36 (19 – 80) | NA | NA |
|  | Capture =0.2 | 15.5 ± 2.2 (13 – 20) | 17.6 ± 2.9 (15 – 26) | 20.1 ± 3.7 (13 - 20) | 23.2 ± 4.9 (18 – 42) | 27.0 -± 6.5 (19 – 62) | 31.9 ± 8.8 (21 – 81) |
|  | Capture =0.3 | 15.1 ± 1.3 (14 – 18) | 16.6 ± 0.9 (16 – 20) | 18.3 ± 2.0 (17 – 23) | 20.1 ± 2.5 (18 – 25) | 22.1 ± 3.0 (20 – 29) | 24.5 ± 3.7 (21 – 33) |
|  | Capture =0.4 | 16.6 ± 0.9 (15 - 17) | 17.9 ± 1.1 (16 – 18) | 19.2 ± 1.3 (18 – 20) | 20.6 ± 1.5 (19 – 21) | 22.1 ± 3.0 (20 – 29) | 24.5 ± 3.7 (21 – 33) |
| *Occasions = 15* | |  |  |  |  |  |  |
|  | Capture = 0.15 | 17.5 ± 1.4 (13 – 19) | 18.9 ± 1.7 (15 – 21) | 20.6 ± 2.1 (16 – 42) | 22.3 ± 2.5 (18 – 27) | 24.2 ± 2.9 (20 – 31) | 26.3 ± 3.4 (21 – 35) |
|  | Capture = 0.3 | 16.4 ± 0.7 (15 – 16) | 17.6 ± 0.8 (16 – 17) | 18.8 ± 1.0 (17 – 19) | 20.0 ± 1.1 (18 – 20) | 21.3 ± 1.3 (19 – 21) | 22.7 ± 1.4 (20 – 24) |
| *Occasions = 30* | |  |  |  |  |  |  |
|  | Capture = 0.15 | 16.2 ± 0.4 (15 – 16) | 17.2 ± 0.5 (16 – 18) | 18.3 ± 0.6 (17 – 19) | 19.4 ± 0.7 (18 – 20) | 20.6 ± 0.8 (19 – 21) | 21.8 ± 1.0 (20 – 23) |
|  | Capture = 0.40 | 16 ± 0.01 (16 – 16) | 17 ± 0.01 (17 – 17) | 18 ± 0.01 (18 – 18) | 19 ± 0.01 (19 – 19) | 20 ± 0.02 (20 – 20) | 21 ± 0.02 (21 – 21) |

**Supplementary Fig. S1**. The relationship between the probability of making an error when classifying a capture event and the number of times that capture event was excluded by other observers. The line shows the mean expectation from a Bayesian binomial model (see model eq. 2 from Appendix S2 below) and the shaded area the 95% Credible Intervals.


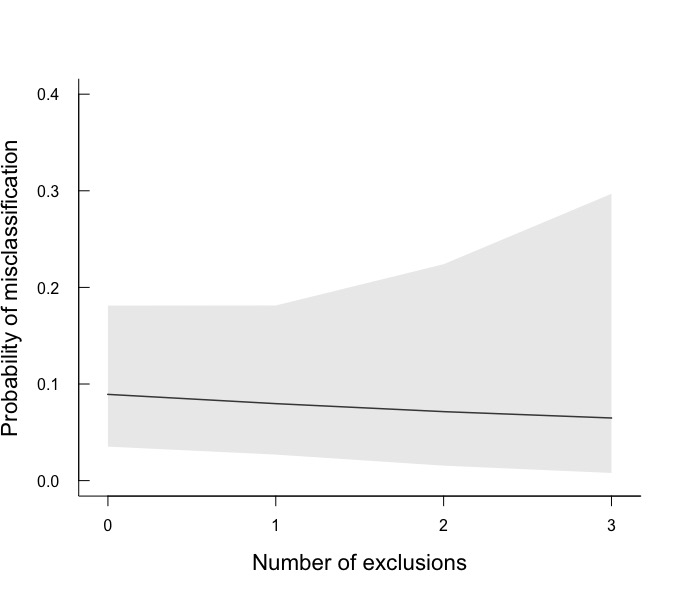


**Appendix S1** Protocol for Observers: this is the background and instructions given to the 8 observers who classified the camera-trap photos

Estimating the accuracy of snow leopard (*Panthera* *uncia*) identification using remote triggered cameras: Instructions for the observers

**Background and purpose**

Camera traps are widely used as a method to survey populations of species that are cryptic, inhabit inaccessible areas or occur in low numbers. One prerequisite for using this method is that it is possible to identify the individuals through physical features, for example spot patterns in the fur. In the case of identification of snow leopard individuals, much effort is spent trying to identify pictures that are blurry, dark or where only a small part of the snow leopard is visible. To our knowledge, no tests have been made to evaluate the accuracy of identification of trap camera pictures and most certainly this has not been made for snow leopards. The aim is to determine how accurate the method is.

**Method description**

You have received pictures from an unknown number of snow leopards, all of which include photos from the animal’s right side. The pictures are organized in series of 3-11 pictures from one photo event (i.e. a series of photos from the same cat and the same photo event). For each snow leopard the number of series it appears in varies from one and upwards.

Pictures are sorted into 40 folders with one series per folder (e.g. series 1, series 2 etc).

Your task is to identify which series of pictures come from the same cat. Please give each cat a unique letter staring with Cat A, then Cat B, etc, and then list the series that you think belong to that cat. Attached to this e-mail is a form for reporting your results (protocol for part one). If you find a series too difficult to use for identification, place it in the category “Series not possible to ID”. **Please put in the same effort as you do when working with pictures from the wild to make this study relevant for evaluating the accuracy of the technique.** The pictures have been sent to eight different observers, you should identify the pictures on your own, meaning you should not co-operate during the identification process.

Please send your results to XXXX who will provide each observer with a random ID and send the protocols to XXXX who in turn will give you a new ID; this will ensure that you will remain anonymous. If you have any questions, contact XXXX at XXXX

**Appendix S2**. Bayesian binomial model for estimating classification error rates based on observer expertise (n = 8 indexed to i). For each type of error (e.g. exclusion, split, combine) separate models were run with the number of specific classification errors specified for that analysis. The number of classification attempts was the number of capture events considered by the observer. Thus for exclusion errors, the number of classification attempts = 40. For other types of error, the number of classification attempts was 40 - number of capture events excluded. For estimates where we were interested in the total number of errors (regardless of observer expertise), the deterministic equation was dropped from the model (line 2) and p was estimated directly using a minimally-informative prior (Beta(1,1)). For estimating the expected credible ranges of observer error, we simulated data based on the model parameters [i.e. sim.expected ~ Binomial(attempts, p)].

model equation 1:


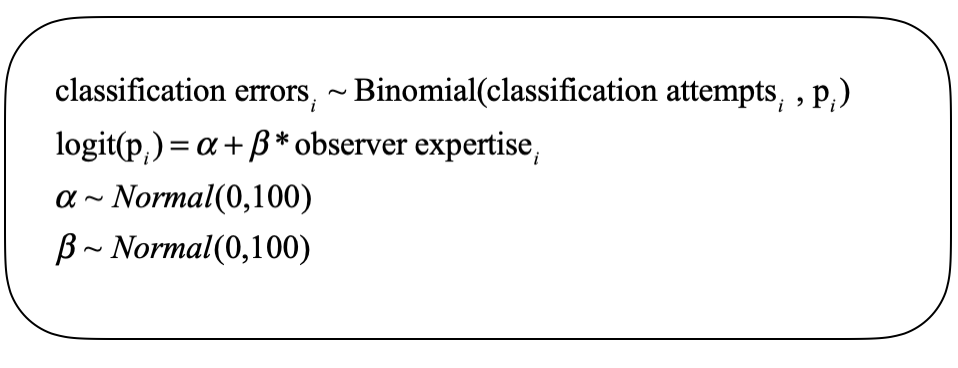


**Appendix S3.** Hierarchical Bayesian binomial model examining the factors influencing misclassification of individual capture events (capture event errors) containing camera-trapped photo sequences (n = 40). Here the number of errors per capture event (maximum = 8 for the eight observers classifying the capture events) is related to the total number of times the capture event was given an individual identity (i.e. was classified, not excluded) by observers multiplied by the probability of an error (p) within a Binomial likelihood. The probability of error (p) was related to the number of times the capture event was excluded (i.e. were capture events that tended to be excluded more often, more difficult to classify correctly? - this was the main parameter of interest in this analysis as it indicates whether the exclusions were ‘true’ exclusions - in that observers exclude the capture events rather than classifying it because they believe classification would result in an error) and the total number of capture events belonging to an individual cat.

The probability of capture event errors was also adjusted based on the identity of the individual cat whose encounter history the capture event belonged to (the random effect on the intercept of the deterministic equation). This hierarchical level on the intercept was necessary to account for overdispersion in the observations. Indexing relates to: i=number of capture events (40) and j=number of cats (16). Priors were minimally informative.

model equation 2:


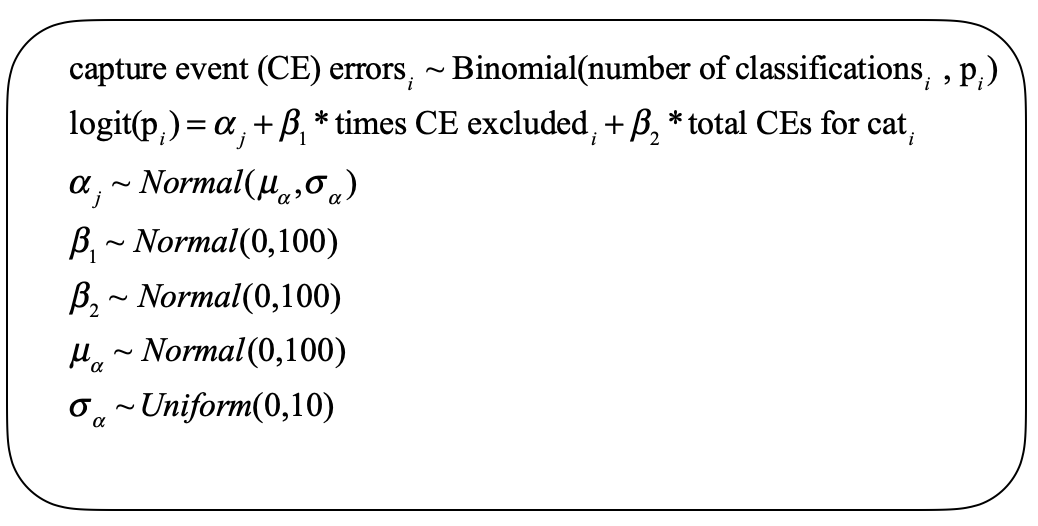


**Appendix S4.** For estimating population size from each observer’s capture history we used the closedp() function from the R package ’Rcapture’ (Baillargeon & Rivest 2007). This method compares different loglinear model estimators while accounting for heterogeneity in capture probability between individuals (constant, Chao, Darroch and Poisson2 heterogeneity models (Rivest & Daigle 2004))

We also created R code for creating simulated encounter histories to demonstrate the effect of splitting errors on population estimates within a capture-recapture analytical framework. For the simulations, this code was surrounded by loops to vary the encounter history (EH) parameters across different values, as well as 1000 repetitions for each EH setting to derive estimates of mean expected population estimates and their errors. In addition, each EH was corrupted by 1 splitting error after each set of simulations (up to 5 splits) to simulate splitting errors and to estimate their effect on population abundance under different EH scenarios (see Fig 3 & Table S2). We used the same capture-recapture modeling framework as used to determine population abundance estimates for each observer in the main paper (i.e. ‘Rcapture’); however for simulations we only retrieved estimates from the ‘constant’ heterogeneity model (m0) for comparison between simulations. If convergence errors occurred, those individual estimates were discarded. From each run of simulations, we report the median, standard deviation and the 95% confidence interval of the abundance estimates generated. Note that for some combinations of capture probability and capture occasions in the second set of simulations, only a limited number of splitting errors could be examined (see Table S2); this was because some capture histories did not have enough resightings to allow splitting errors.

##R code for the simulation runs

#1. set parameters for creating the encounter history (EH)

occ<-7 #number of capture occasions

n<-16 #true population size

p<-0.2 #probability of detection

total<-numeric(occ*n) #vector to hold total observations

#2. create the encounter history (EH) for all animals

for(i in 1:length(total)){

sample<-runif(1,0,1) #take random sample between 0 & 1

total[i]<-ifelse(sample<p,1,0) #decide whether an animal 'observed' or not

} #close i loop

obs.matrix<-matrix(total, ncol=occ, nrow=n) #use ‘total’ vector of 0's and 1's to create encounter matrix

#note that the encounter history includes rows that are all zeros (i.e. animal not observed)

#3. remove rows where no observations occurred for that animal (i.e. present but undetected) to create EH that looks like one you'd have from the field

seen<-numeric(nrow(obs.matrix))

for(i in 1:nrow(obs.matrix)){

seen[i]<-ifelse(sum(obs.matrix[i,])==0,0,1) #for each row, which have only 0's

} #close i loop

obs.matrix.seen<-obs.matrix[seen==1,]

#4. run the closed capture-recapture model to estimate population size from the EH using the Rcapture package

library(Rcapture)

catch<-closedp.0(obs.matrix.seen)

est.1<-catch$results[1,1] #for the pop estimate for M0

sd<-catch$results[1,2] #for the pop estimate SD for M0

**Appendix S5.** Example of a simulated encounter history based on 16 animals in the population (rows), 7 encounter occasions (columns) and a probability of (re)capture of 0.2 (which determines whether at each occasion the animal is seen (1) or not (0). Note that some animals in this encounter history are not seen at all (all 0’s which would not be represented in a real encounter history because their presence would be unknown). Thus, it is the ratio of 0’s to 1’s in the animals that are seen that determines the detection probability and therefore the possibility that unseen animals are present in the population. It should be noted that a splitting error not only adds a new animal to the known population, but also increases the ratio of 0’s to 1’s in the animals that are seen (see example below the EH). This will reduce the estimates of (re)capture probability and inflate estimates of the number of unseen animals in the population. It is by these two routes that splitting errors inflate population abundance estimates.

[,1] [,2] [,3] [,4] [,5] [,6] [,7]

[1,] 0 0 0 0 0 0 0

[2,] 1 0 0 0 1 0 1

[3,] 0 0 0 0 1 0 0

[4,] 0 0 0 0 0 0 0

[5,] 0 0 0 0 0 0 0

[6,] 0 0 0 0 1 0 0

[7,] 0 1 0 1 0 0 0

[8,] 0 1 0 0 0 0 0

[9,] 0 0 0 0 0 0 0

[10,] 0 0 0 0 0 1 1

[11,] 1 0 0 1 0 0 0

[12,] 0 0 0 0 0 0 1

[13,] 0 0 0 0 0 0 0

[14,] 0 0 1 0 0 0 0

[15,] 0 0 0 1 1 0 0

[16,] 1 0 1 0 0 0 0

Example of splitting error on the third occasion of an individual animal’s EH

1 0 1 0 0 0 1

Becomes

1 0 0 0 0 0 1

0 0 1 0 0 0 0

Here we not only have the addition of another individual in the EH, but the addition of another 7 0’s in total. These 0’s will inflate the estimates of (re)capture probability and further inflate the population estimate.

**References.**

Baillargeon, S. & Rivest, L. (2007). Rcapture: loglinear models for capture-recapture in R. *Journal of Statistical Software*, 19, 1–31.

Rivest, L.P. & Daigle, G. (2004). Loglinear Models for the Robust Design in Mark–Recapture Experiments. *Biometrics*, 60, 100–107.
